# Nanostructured Titanium-Based Support Modulates Endothelial and Mesenchymal Cell Communication Through Extracellular Vesicles

**DOI:** 10.3390/ijms27219972

**Published:** 2026-11-06

**Authors:** Maria Pia Cavaleri, Yasar Kemal Erdogan, Luna Ardondi, Giulia Brunello, Lucia Sileo, Ilenia Pia Cappucci, Laura Basile, Francesca Zalunardo, Sivolella Stefano, Gökhan Polat, Letizia Ferroni, Barbara Zavan

**Affiliations:** 1 Department of Medical Sciences, University of Ferrara, 44121 Ferrara, Italyluna.ardondi@unife.it (L.A.);; 2 Department of Biomedical Engineering, Isparta University of Applied Science, 32040 Isparta, Turkey; 3 Biomedical Engineering Program, Middle East Technical University, 06800 Ankara, Turkey; 4 Department of Oral Surgery, University Hospital Düsseldorf, 40225 Düsseldorf, Germany; giulia-bru@libero.it; 5 Department of Orthodontics and Dentofacial Orthopedics, Charité-Universitätsmedizin, 14197 Berlin, Germany; 6 Department of Neuroscience, Dentistry Section, University of Padova, 35128 Padova, Italy; 7 IRCCS Maria Cecilia Hospital, GVM Care & Research, 48033 Cotignola, Italy; 8 Department of Metallurgical and Materials Engineering, İzmir Katip Celebi University, 35620 İzmir, Turkey

**Keywords:** nanostructuration, extracellular vesicles, endothelial cells, osteogenesis, angiogenesis

## Abstract

Titanium surface nanotopography can regulate cellular behavior not only through direct cell–material interactions but also by influencing extracellular vesicle (EV)-mediated communication. Here, we investigated how anodized nanostructured Ti–Nb–Ta–Zr–Hf surfaces affect human endothelial cells and human adipose-derived stem cells (hADSCs), as well as the biological activity of EVs released by each cell type. Nanostructuring increased surface roughness and hydrophilicity while maintaining compatibility with both cellular populations. Endothelial cells progressively adapted to the titanium surfaces, maintaining viability and *NOS3* expression while reducing inflammatory, proliferative, and angiogenic activation markers. Although surface topography did not substantially affect endothelial EV size or release, EVs generated on nanostructured titanium displayed increased CD81 expression, enhanced recipient-cell and preserved endothelial monolayer organization compared with EVs generated on the bare surface. In hADSCs, the nanostructured interface induced a rounded morphology associated with reduced expression of focal-adhesion and cytoskeletal-tension mediators, including *VCL*, *PTK2*, *ACTA2*, and *ROCK1*, together with downregulation of the mechanosensitive transcriptional regulators *YAP1* and *WWTR1*/*TAZ*. This response was accompanied by upregulation of the pro-osteogenic miRNAs miR-21-5p and miR-20a-5p, downregulation of the osteogenesis-inhibitory miRNAs miR-133a-5p and miR-138-5p, and activation of *RUNX2*, *ALPL*, *BGLAP*, and *SPARC*. hADSC-derived EVs were efficiently internalized by recipient cells and promoted a concordant osteogenic-priming response. Overall, nanostructured titanium modulates direct cellular mechanotransduction and the biological activity of EV-mediated signals in a cell-type-specific manner. Rather than demonstrating osteogenic–angiogenic coupling, these findings reveal parallel endothelial homeostatic and mesenchymal osteogenic responses that may independently support the establishment of a regenerative peri-implant microenvironment.

## 1. Introduction

In recent years, nanostructured surfaces have emerged as a powerful strategy to modulate cell behavior by recapitulating key features of the native extracellular matrix [1,2]. By influencing protein adsorption, cell adhesion, and mechanotransduction pathways, nanoscale topographies provide a means to actively direct biological responses rather than serving as passive support [3]. Titanium and its alloys are extensively used due to their high strength-to-weight ratio, corrosion resistance, and established biocompatibility [4]. However, the biological performance of titanium implants is determined not only by their bulk properties but also, and critically, by the physicochemical characteristics of their surfaces. Surface topography, roughness, chemistry, and surface energy collectively define the initial interactions between titanium and the biological environment, ultimately influencing tissue integration and regenerative outcomes [5,6]. Advances in surface engineering have enabled the fabrication of titanium surfaces with well-defined nanoscale features through techniques such as anodization, acid etching, sandblasting, laser processing, and additive manufacturing-based post-treatments [7,8]. These approaches allow for precise control over nanoscale roughness, wettability, and surface energy, parameters known to regulate protein adsorption and early cell adhesion. Nanostructured titanium surfaces more closely resemble the hierarchical architecture of bone extracellular matrix, thereby providing instructive physical cues that modulate cell behavior via mechanotransduction pathways [2,9].

While conventional osseointegration models view these physical cues as direct cell-autonomous drivers of line age differentiation, a profound paradigm shift demonstrates that the impact of nanostructured surfaces extends beyond direct cell–biomaterial contact, acting as a potent regulator of intercellular communication [10,11]. By inducing specific shifts in cell morphology and cytoskeletal tension, nanoscale topographies can effectively reprogram the cellular secretome, including the release of soluble factors and extracellular vesicles (EVs) [10,12]. These EVs have emerged as fundamental mediators of tissue regeneration, serving as sophisticated delivery vehicles that encapsulate a diverse repertoire of proteins, lipids, and nucleic acids [13]. Unlike conventional paracrine signaling via free-floating ligands, the EV membrane protects its bioactive cargo from enzymatic degradation, ensuring the targeted and stable delivery of signals required to coordinate the complex crosstalk between osteogenic and endothelial compartments [14,15,16].

This material-to-secretome axis operates via strict geometric causality: altering the nanoscale topography provides predictable, direct control over EV biogenesis kinetics and endogenous molecular sorting. When donor cell populations, such as bone marrow mesenchymal stem cells (BMSCs) or macrophages, adhere to highly ordered titania nanotube arrays (TNAs) or micro/nano-hierarchical textures, the restricted cellular spreading and altered focal adhesion clustering trigger intracellular mechanosensitive cascades. This mechanical stress upregulates the small Rab GTPase Rab27b and sphingomyelin phosphodiesterase 2/3 (SMPD3), molecular switches that govern the transport, docking, and exocytosis of late endosomal multivesicular bodies (MVBs) at the plasma membrane, driving a high-yield surge in EV secretion [10,17,18]. Concurrently, specific surface features (e.g., increasing TNA diameters from 25 nm to an optimized 80–95 nm) systematically alter the intracellular cargo loading machinery, inducing the selective encapsulation of a distinct profile of microRNAs (miRNAs). Optimized nanostructured surfaces specifically enrich their derived EVs with pro-angiogenic and osteoinductive master-regulators, most notably miR-21 and miR-3473e [18,19,20].

Crucially, bone regeneration at the implant interface is tightly coupled with vascularization, requiring coordinated osteogenic and angiogenic responses. Nanoscale surface roughness and increased surface energy have been shown to enhance osteoblast differentiation while simultaneously influencing endothelial cell adhesion, migration, and organization. Such surface-driven effects suggest that nanostructured titanium can promote a favorable microenvironment for coupled bone and vascular regeneration, which is essential for stable osseointegration [21,22,23]. Within this microenvironment, the surface-primed EVs act as the primary horizontal vehicle crossing the peri-implant extracellular space to engage the neighboring endothelial compartment. Upon internalization by recipient endothelial cells, these miRNA-laden EVs suppress negative intracellular regulators to directly activate downstream pro-survival and migratory cascades, specifically the PI3K/Akt signaling pathway. This causes a robust stimulation of endothelial cell migration, proliferation, and rapid capillary-like tube formation, functionally coupling early vascularization with matrix mineralization [18,19,24].

The ability to design titanium surfaces that actively promote this osteogenic–endothelial coupling is particularly relevant for clinical conditions characterized by impaired healing, such as diabetes and other inflammatory chronic diseases. In these compromised settings, localized ischemia, reduced vascularization, altered cell signaling, and delayed bone regeneration typically compromise implant success [25]. Nanostructured, high-surface-energy titanium interfaces help overcome these clinical limitations by restoring pro-regenerative cellular responses through the targeted activation of this surface-to-endothelial paracrine pathway [21,26]. Consequently, surface nanotopography is transforming from a simple structural interface into an active biofabrication platform capable of driving cell-free, vesicle-mediated vascularized osseointegration [10,27].

While current literature has predominantly focused on how titanium surface engineering dictates the autonomous behavior of bone-forming cells, the underlying mechanisms of surface-driven paracrine signaling remain critically underexplored [10,28]. To bridge this knowledge gap, the present study systematically investigates how bare and anodized nanostructured titanium surfaces reprogram the regenerative function of cell-derived EVs, establishing a previously unappreciated bridge linking surface nanotopography to local tissue orchestration. Distinct from conventional studies that typically examine isolated cell lineages [10,20], we strategically evaluated the EV secretome from two critical cell types driving clinical osseointegration: human umbilical vein endothelial cells (HUVECs) and adipose-derived stem cells (hADSCs). Our approach fundamentally diverges from existing literature in both its phenotypic observations and its mechanistic focus. In current biomaterial frameworks, surface modifications are often assumed to alter the physical properties—such as the size distribution, concentration, or release kinetics—of the cell-secreted vesicles [17,29,30]. Contrarily, our findings reveal a compelling phenomenon: nanostructured titanium topographies do not substantially alter EV size or release yield, yet they profoundly rewire their intrinsic biological activity. This demonstrates that the biomaterial interface drives a selective biochemical sorting of internal molecular cargo without triggering macroscopic changes in vesicle biogenesis. Through this study, we aim to demonstrate that EVs harvested from cells grown on nanostructured titanium surfaces function as instructive paracrine vectors capable of shifting neighboring recipient cells toward a homeostatic and highly regenerative phenotype. Specifically, we validate that these surface-reprogrammed EVs significantly upregulate cellular activity, accelerate wound healing kinetics, and shift recipient cells toward a vascular homeostatic state. By decoupling vesicle physical morphology from its underlying biological potency, this work establishes a novel conceptual framework for osseointegration, proving that titanium nanostructuring can be exploited as a deterministic tool to engineer highly potent, cell-free, vesicle-mediated therapeutic microenvironments.

## 2. Results

### 2.1. Surface Characterization of Anodized and Non-Anodized Biomaterials

**Figure 1:**
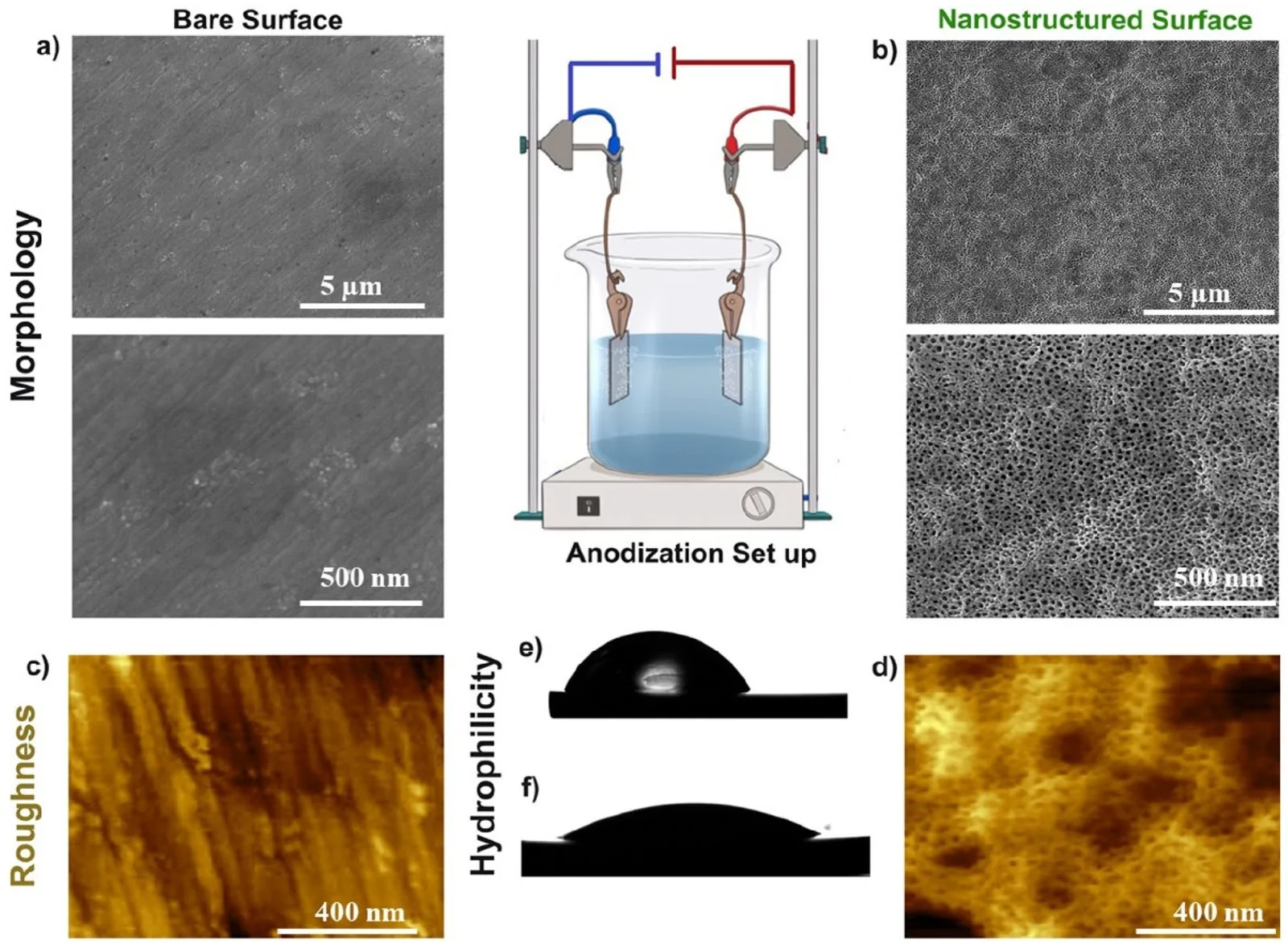
Surface characterization of TNTZ-Hf titanium-based alloys. (**a**,**b**) Representative scanning electron microscopy (SEM) images, (**c**,**d**) atomic force microscopy (AFM) topographic maps, and (**e**,**f**) water contact angle profiles of bare and nanostructured surfaces, illustrating differences in morphology, roughness, and hydrophilicity.

**Figure 2:**
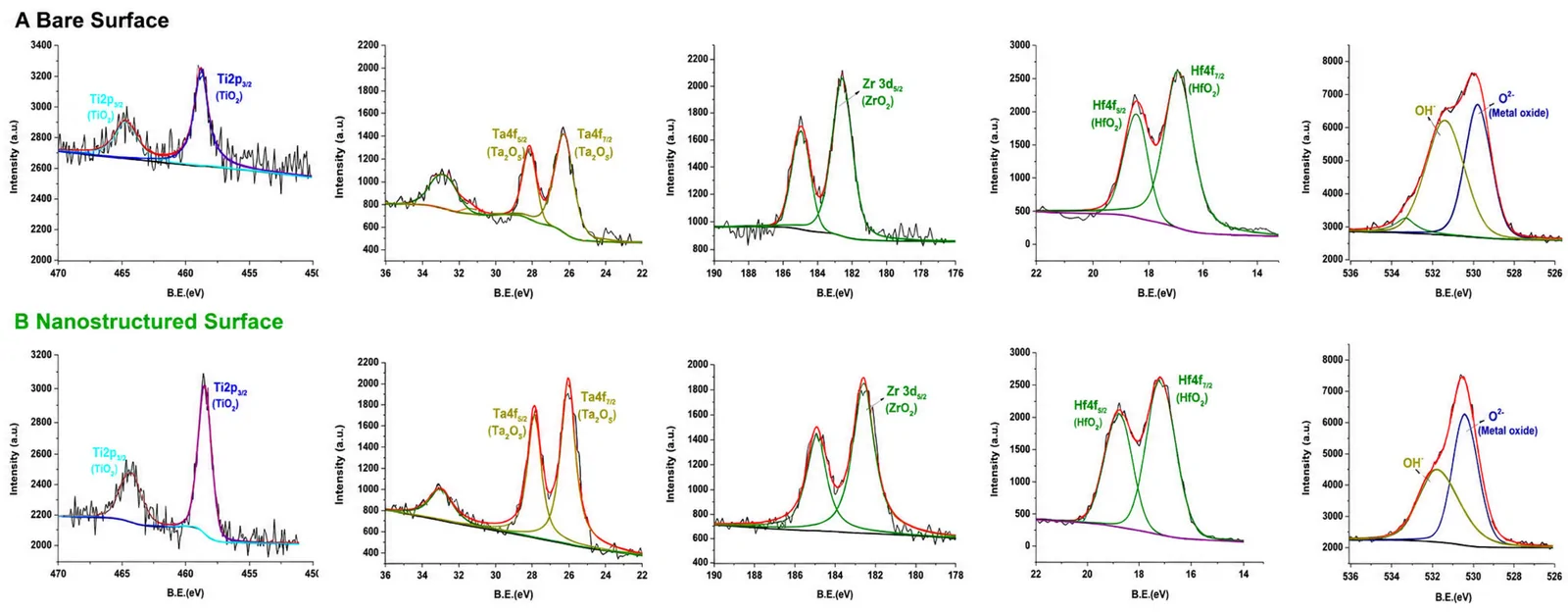
Representative high-resolution XPS spectra of (**A**) bare and (**B**) nanostructured TNTZ-Hf surfaces showing the Ti 2p, Ta 4f, Zr 3d, Hf 4f, and O 1s core-level regions.

Scanning electron microscopy (SEM) and atomic force microscopy (AFM) revealed marked morphological distinctions between the two titanium-based surfaces (Figure 1). While the bare surface exhibited a smooth, featureless topography characterized by machining marks and an absence of porosity (Figure 1a), the nanostructured surface displayed a highly uniform layer of well-defined nanopores (Figure 1b) with an average diameter of 24.5 ± 2.1 nm. AFM quantitative analysis confirmed this structural transformation, with the mean surface roughness (Ra) increasing significantly from 2.42 ± 0.32 nm on the bare surface to 12.48 ± 0.55 nm on the nanostructured surface (AFM representative images in Figure 1c and Figure 1d, respectively). This topographic evolution was accompanied by a dramatic shift in surface energy; the water contact angle transitioned from 71.2 ± 4.9° (bare) to a highly hydrophilic 21.6 ± 4.5° (nanostructured). The observed change in contact angle indicates a modification of surface wettability. The chemical basis for these changes was further elucidated by high-resolution XPS (Figure 2). The core-level spectra for Ti 2p, Ta 4f, Zr 3d, Hf 4f, and O 1s confirmed the presence of a stable, multi-component oxide layer on both surfaces. The absence of metallic peaks across all spectra indicates that both surfaces are covered by a dense, uniform passive film that effectively masks the underlying metallic substrate. Crucially, the nanostructuring process preserved the chemical integrity of the alloy, as the peak positions for the modified surface remained aligned with those of the bare control. Further deconvolution of the O 1s region identified two primary oxygen environments: lattice oxygen (O2-) within the metal oxide framework and surface hydroxyl groups (–OH) derived from chemisorbed water. The XPS profile of the nanostructured surface showed a comparatively greater contribution in the spectral region associated with oxygen-containing and hydroxyl-related species. Although their relative abundance was not quantified by peak deconvolution, this qualitative observation is consistent with the independently measured increase in surface wettability.

### 2.2. Impact of Nanostructured Surfaces on Endothelial Cells

**Figure 3:**
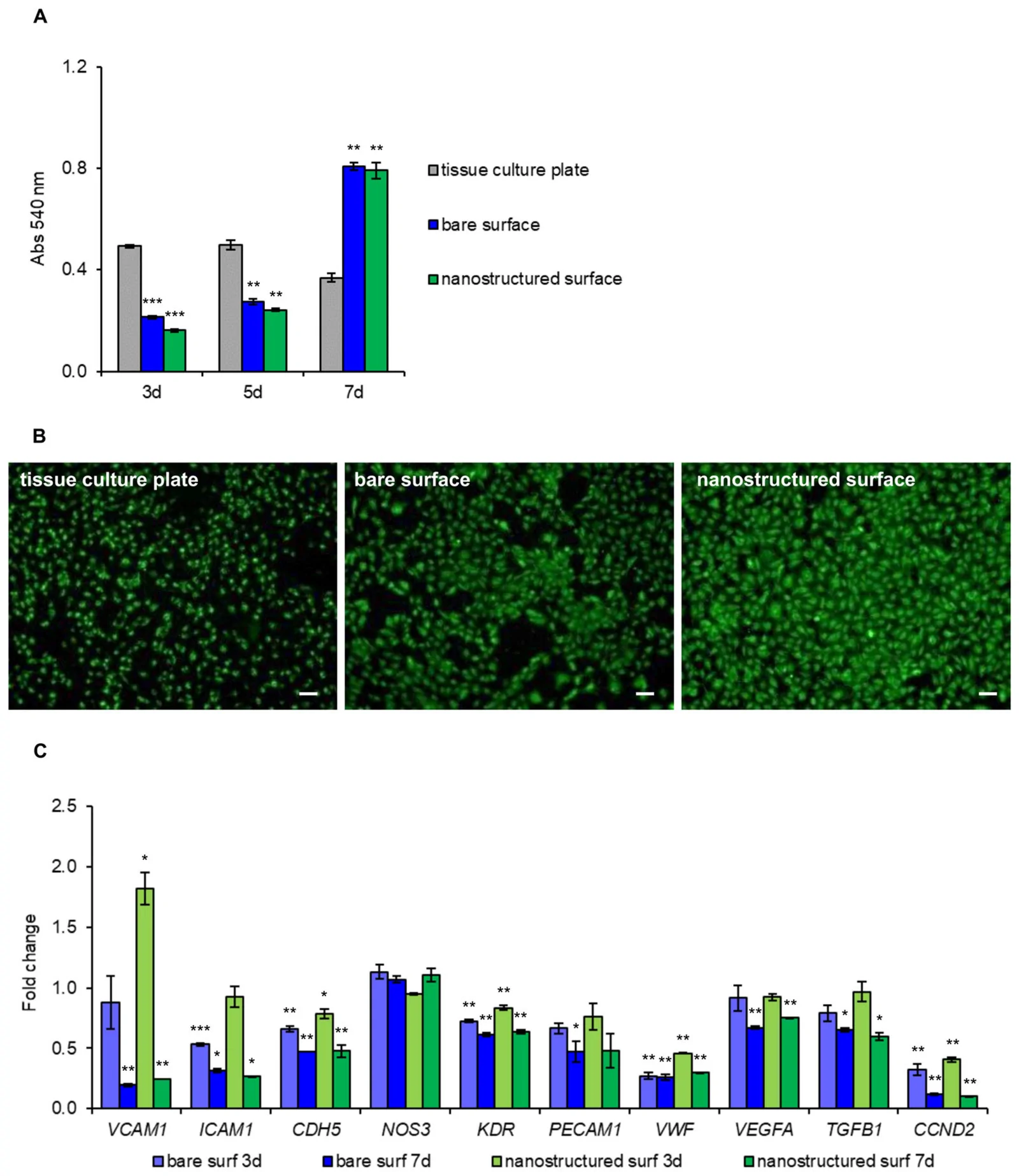
Human endothelial cells cultured on bare and nanostructured surfaces. (**A**) Cell activity assessed by MTS assay after 3, 5, and 7 days. Data are presented as mean ± SD from three independent biological experiments (*n* = 3), with technical triplicates averaged before statistical analysis. (**B**) Representative Calcein-AM staining images showing cell viability and morphology. (**C**) Relative gene expression profile after 3 and 7 days. Gene expression was normalized using the geometric mean of *ACTB* and *GAPDH*. Data are presented as mean ± SD (*n* = 3). Statistical analysis was performed using one-way ANOVA followed by Bonferroni’s post hoc test. * *p* < 0.05; ** *p* < 0.01; *** *p* < 0.001.

Endothelial cell activity was assessed at 3, 5, and 7 days of culture on bare and nanostructured titanium surfaces, using tissue-culture plate (TCP) as the reference condition (Figure 3A). At day 3, cells cultured on TCP showed significantly higher MTS values than cells cultured on either titanium surface. The same pattern was observed on day 5. Between days 5 and 7, MTS values increased in cells cultured on both bare and nanostructured titanium. On day 7, no statistically significant difference was observed between the two titanium surfaces. These results demonstrate a time-dependent variation in endothelial activity according to the culture substrate, with lower values on titanium during the initial culture period and increased activity at the later time point. Calcein-AM staining was performed to complement the MTS analysis and to visualize active cells on the different substrates (Figure 3B). Green fluorescence was detected in cells cultured on TCP and on both titanium surfaces, confirming intracellular conversion of Calcein-AM by viable cells. The images also showed adherent and spread endothelial cells under all experimental conditions, although differences in cell distribution and organization were observed among the substrates. These images provide qualitative information on cell presence, morphology and spatial distribution but were not used to infer endothelial barrier integrity or functional maturation. To determine whether the culture substrate was associated with changes in endothelial gene expression, a panel comprising *VCAM1*, *ICAM1*, *CDH5*, *PECAM1*, *VWF*, *NOS3*, *KDR*, *VEGFA*, *TGFB1*, and *CCND2* was analyzed at days 3 and 7 (Figure 3C). These genes were selected because of their established involvement in endothelial adhesion, cell–cell junctions, endothelial identity, growth-factor signaling, nitric oxide synthesis, extracellular-matrix regulation and cell-cycle progression. Their expression was used to characterize the transcriptional response to the surfaces and not as a direct functional measurement of inflammation, angiogenesis, barrier integrity or endothelial maturation. At day 3, *VCAM1* expression was significantly increased in cells cultured on the nanostructured surface and reduced in cells cultured on the bare surface relative to TCP. *ICAM1* was significantly downregulated on the bare surface, whereas its expression on the nanostructured surface remained closer to that observed on TCP. The expression of *CDH5* and *PECAM1* was moderately reduced on both titanium surfaces, while *VWF* was significantly downregulated under both conditions. *NOS3* expression did not differ significantly among the experimental groups. *KDR* was significantly downregulated on both titanium surfaces, whereas no significant differences were detected for *VEGFA* or *TGFB1*. *CCND2* expression was also significantly reduced on both bare and nanostructured titanium. At day 7, *VCAM1* and *ICAM1* expression was reduced on both titanium surfaces compared with TCP. Similarly, *CDH5*, *PECAM1*, and *VWF* remained significantly lower than in the TCP group. The expression of *KDR, VEGFA*, and *CCND2* was also significantly reduced on both titanium surfaces, whereas *NOS3* remained comparable among the experimental conditions. These data identify a time- and substrate-dependent modulation of the endothelial transcriptional profile. However, the observed gene-expression changes alone do not establish corresponding alterations in inflammatory activity, angiogenic capacity, barrier function or endothelial maturation. Overall, both titanium surfaces supported the presence of active endothelial cells throughout the experimental period and induced distinct temporal changes in activity and gene expression compared with TCP. The nanostructured surface produced a specific early transcriptional response, particularly involving *VCAM1*, while many of the changes observed at day 7 were shared by the bare and nanostructured surfaces. These findings demonstrate that titanium surface properties modulate endothelial cellular and molecular responses, without establishing a specific functional effect on angiogenesis, vasculogenesis or endothelial barrier formation.

**Figure 4:**
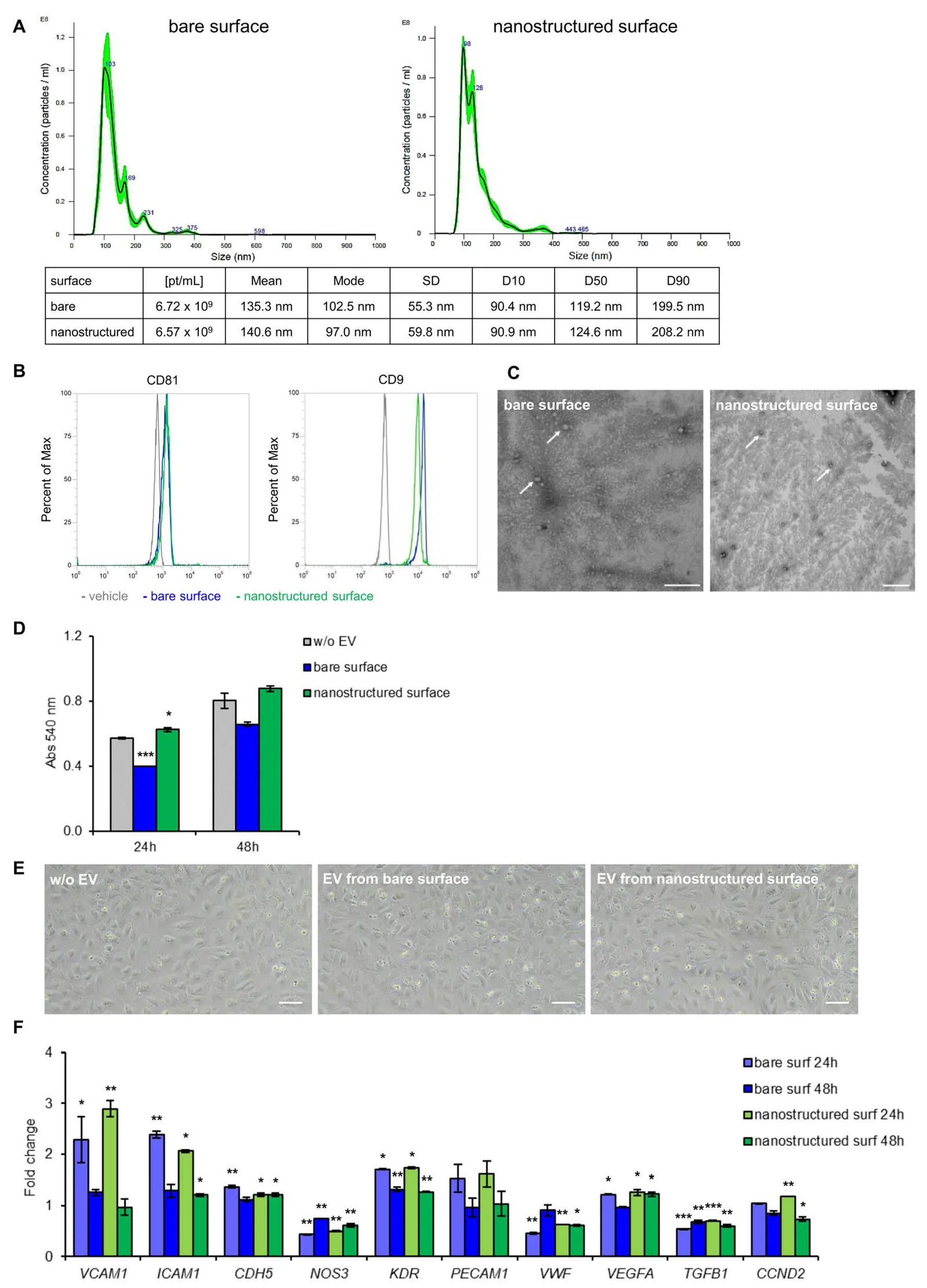
Characterization and functional assessment of endothelial cell-derived EVs. (**A**) Particle size distribution and concentration. (**B**) Flow cytometric analysis of EV markers. (**C**) Representative TEM images of EVs from endothelial cells cultured on bare and nanostructured surfaces, respectively. White arrows indicate representative EVs. Scale bars: 200 nm (bare) and 500 nm (nanostructured), respectively. (**D**) Activity and (**E**) representative morphology of recipient endothelial cells after EV treatment. (**F**) Gene expression profile of recipient endothelial cells. Quantitative data are presented as mean ± SD from three independent biological experiments (*n* = 3). Statistical analysis was performed using one-way ANOVA followed by Bonferroni’s post hoc test. * *p* < 0.05; ** *p* < 0.01; *** *p* < 0.001.

EVs were isolated from the conditioned media of endothelial cells cultured on both bare and nanostructured surfaces (Figure 4). Characterization via nanoparticle tracking analysis (NTA) revealed consistent secretion levels across both groups, yielding particle concentrations of 6.72 × 10^9^ particles/mL for the bare surface and 6.57 × 10^9^ particles/mL for the nanostructured surface (Figure 4A). The mean EV size was slightly higher for the nanostructured surface (140.6 nm) compared to the bare surface (135.3 nm), while the modal size was marginally lower (97.0 nm vs. 102.5 nm). Both conditions exhibited similar size distributions, with overlapping D10, D50, and D90 values, indicating no major differences in EV population heterogeneity. EVs were further characterized for the expression of tetraspanin surface markers using bead-based capture followed by flow cytometry (Figure 4B). EVs derived from both bare and nanostructured surfaces showed strong positivity for CD9, with 98.4% and 99.6% of events within the positive gate, respectively. Analysis of CD81 expression revealed a lower but detectable signal compared to CD9. EVs from cells cultured on bare surfaces showed 66.7% CD81-positive events, whereas EVs derived from nanostructured surfaces exhibited an increased CD81 positivity of 78.7%. These results confirm the vesicular nature of the isolated particles and suggest that nanostructured surfaces may modulate EV tetraspanin composition. To elucidate how the nanostructured surface modulates the paracrine activity of endothelial cells, assays based on autologous EV treatment were performed to evaluate activity and functional phenotype (Figure 4D,F). Human endothelial cells were treated with EVs derived from cells cultured on bare or nanostructured surfaces. Cells treated with EVs derived from bare surfaces showed a significant reduction in activity at 24 h compared to untreated controls, with values remaining lower at 48 h and approaching statistical significance (Figure 4D). In contrast, endothelial cells treated with EVs derived from nanostructured surfaces exhibited significantly higher activity compared to the bare-surface EV group. At 48 h, activity further increased and exceeded control levels, although this difference did not reach statistical significance (Figure 4D). These findings were consistent with phase-contrast microscopy observations at 48 h (Figure 4F). Untreated cells formed a typical cobblestone-like monolayer with well-defined cell–cell contacts, indicative of a healthy endothelial phenotype. Cells treated with EVs derived from bare surfaces displayed a reduced monolayer organization. Conversely, endothelial cells treated with EVs derived from nanostructured surfaces maintained a well-spread morphology and preserved monolayer integrity, comparable to or exceeding that of untreated cells. The gene expression profile of endothelial cells treated with autologous EVs derived from bare or nanostructured surfaces was assessed at 24 and 48 h (Figure 4F). At 24 h, EVs from both bare and nanostructured surfaces significantly upregulated the expression of adhesion molecules *VCAM1* and *ICAM1* compared to untreated controls. *KDR* (*VEGFR2*) and *PECAM1* were also upregulated, with statistical significance for *KDR* in both groups. In contrast, *NOS3*, *VWF*, and *TGFβ* were significantly downregulated, particularly with EVs from bare surfaces. Minor changes were observed for *CDH5*, *VEGFA*, and *CCND2*, with moderate statistical significance. At 48 h, the expression of adhesion molecules generally returned closer to control levels for cells treated with nanostructured-surface EVs, whereas EVs from bare surfaces still tended to elevate *ICAM1* and slightly increased *KDR* and *CDH5*. *NOS3*, *VWF*, and *TGFβ* remained downregulated in both EV-treated groups, though the effect was slightly attenuated at 48 h compared to 24 h. Overall, these results indicate that EVs derived from endothelial cells cultured on the two titanium surfaces induced distinct responses in recipient endothelial cells. Compared with EVs generated on the bare surface, EVs derived from the nanostructured surface significantly increased endothelial activity and preserved the characteristic cobblestone-like morphology and organization of the endothelial monolayer. These effects were accompanied by time-dependent modulation of genes associated with endothelial activation, adhesion, intercellular junctions, vascular signaling and cell-cycle regulation, including *VCAM1*, *ICAM1*, *PECAM1*, *CDH5*, *KDR*, *NOS3*, *VWF*, *VEGFA*, *TGFB1*, and *CCND2*. These findings demonstrate a surface-dependent modulation of endothelial activity and phenotype but do not establish the induction of angiogenesis or vasculogenesis.

### 2.3. Impact of Nanostructured and Bare Surfaces on Mesenchymal Stem Cells

**Figure 5:**
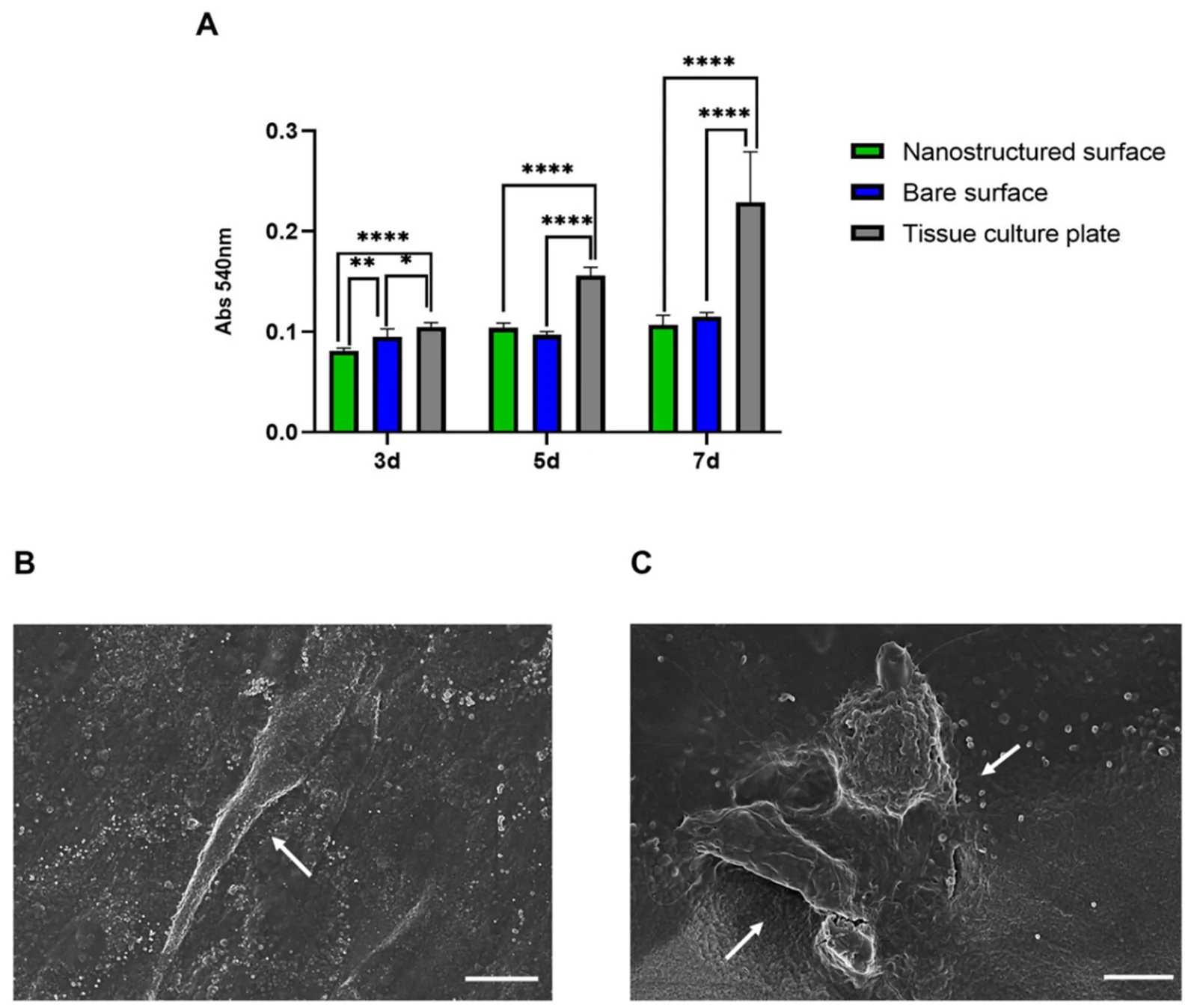
hADSC adhesion and proliferation on bare and nanostructured titanium surfaces. (**A**) Cell activity determined by MTS assay. Data are presented as mean ± SD from three independent biological experiments (*n* = 3). Statistical analysis was performed using one-way ANOVA followed by Bonferroni’s post hoc test. * *p* < 0.05; ** *p* < 0.01; **** *p* < 0.0001. (**B**,**C**) Representative SEM micrographs of hADSC morphology after 3 days of culture. White arrows indicate the cells.

The activity and proliferation of hADSCs cultured on each surface were evaluated via MTS assay at 3, 5, and 7 days (Figure 5A), using TCP as a control. All groups exhibited a progressive increase in absorbance over the seven-day period, indicating sustained cell viability and proliferation on both surfaces. Quantitative analysis showed that both titanium alloy surfaces supported cell growth without inducing cytotoxic effects. A significant decrease in activity on both biomaterials compared to the TCP was observed already at day-3 and persisted throughout the experiment. This reduction is not indicative of cytotoxicity and may reflect the early onset of osteogenic differentiation, consistent with the reduced proliferation typically seen during mesenchymal stem cell commitment, supporting the hypothesized role of the biomaterials. These results indicate that both surface topographies, bare and nanostructured, provide a supportive environment that sustains cell viability and mitochondrial function, while influencing cellular interactions and potentially modulating the initial stages of osteogenic differentiation. These findings were corroborated by the cell morphology observed after 3 days of culture on each biomaterial surface (Figure 5B,C). To evaluate the initial cytocompatibility and topographical adaptation of hADSCs at the biomaterial interface, high-resolution SEM analysis was executed. Our structural observations revealed a striking morphological dichotomy dictated exclusively by the underlying titanium surface architecture. When cultured on bare, machined titanium substrates, hADSCs displayed a conventional, flattened, and highly spread morphology, extending wide lamellipodia and establishing diffuse cell-to-substrate contacts. Conversely, when exposed to anodized nanostructured titanium interfaces, the hADSC population underwent a profound spatial rearrangement, adopting a highly restricted, spherical, and rounded conformation. In modern biomaterial literature, such architectural restriction is well-documented to result from the nanoscale dimensions of the topography, which disrupt continuous integrin clustering and physically restrict cell spreading area. Importantly, this topographical confinement and the resulting rounded cell geometry are not indicative of impaired viability, but rather function as a potent physical cue that alters the intracellular tension of the actin network, effectively biasing stem cell signaling toward an osteogenic fate long before the transition is visually completed.

**Figure 6:**
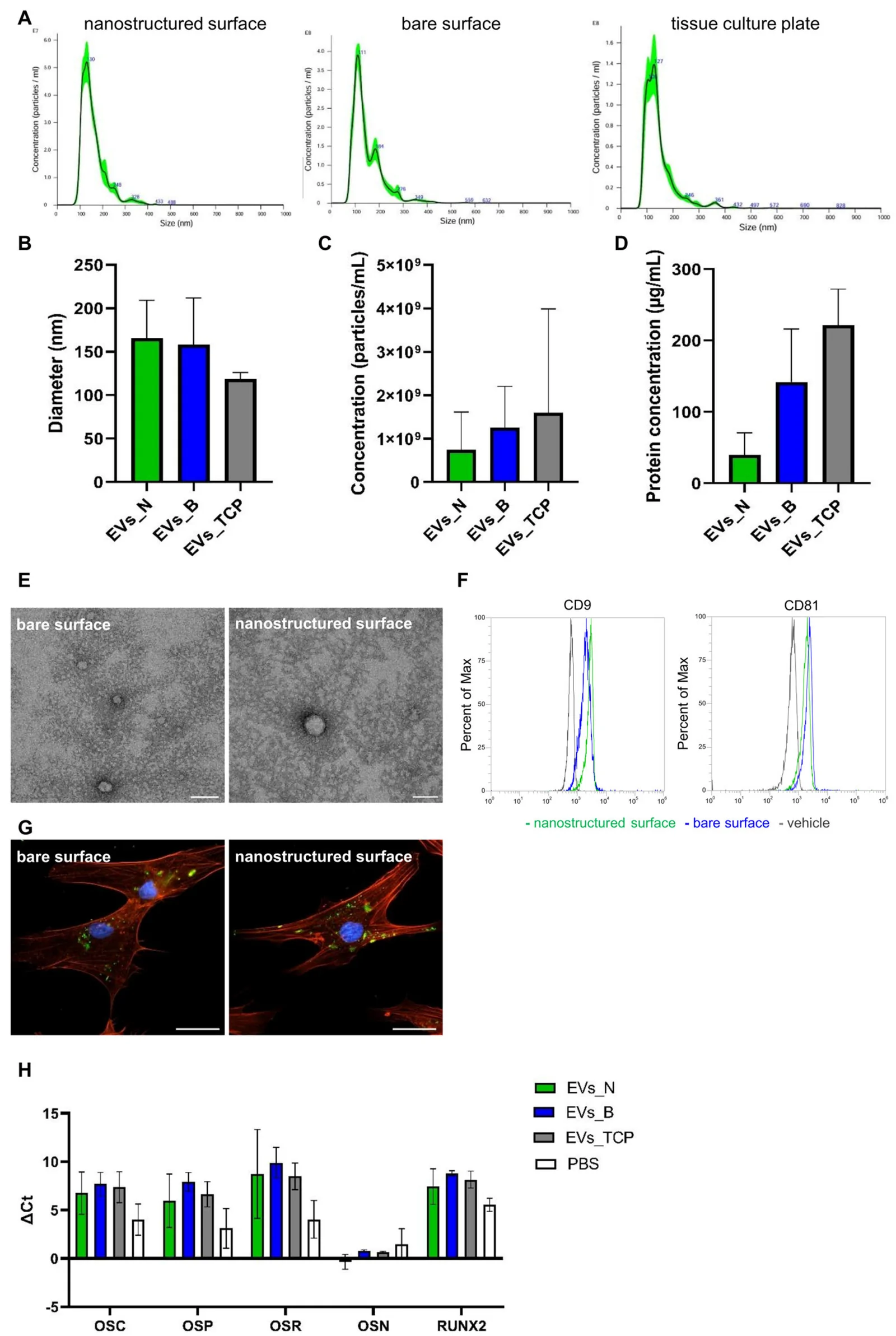
Characterization and functional assessment of hADSC-derived extracellular vesicles. (**A**) Representative size distribution and particle concentration profiles for EVs isolated from nanostructured surfaces, bare surfaces, and tissue culture plate, respectively. (**B**–**D**) Quantitative analysis of EV metrics: (**B**) mean diameter (nm), (**C**) particle concentration (particles/mL), and (**D**) total protein concentration (µg/mL). (**E**) Representative TEM micrographs of EVs derived from bare and nanostructured surfaces; scale bars = 100 nm. (**F**) Flow cytometric analysis of EV markers. (**G**) Fluorescence images demonstrating the cellular internalization of EVs derived from bare and nanostructured surfaces. Green fluorescence indicates localized EV uptake within the cytoplasm; scale bars = 8 µm. (**H**) Relative mRNA expression of key osteogenic markers in recipient hADSCs following EV treatment. No statistically significant differences were observed between experimental groups and the control. EV_N: EV derived from nanostructured surface; EVs_B: EV from bare surface; EVs_TCP: EV from tissue culture plate.

To investigate the influence of surface topography on paracrine signaling, EVs were isolated from the conditioned media of hADSCs cultured on bare and nanostructured surfaces, with TCP as a reference (Figure 6). The physical properties of the isolated vesicles, including size distribution and particle concentration, were quantitatively assessed using NTA. As shown in Figure 6A, the size distribution profiles remained consistent across all three experimental groups, suggesting that the surface modification of the titanium alloy does not fundamentally alter the physical dimensions of the secreted vesicles. As illustrated in Figure 6B, EV diameters across all groups were predominantly distributed within the 100–200 nm range. No statistically significant differences in particle diameter or concentration were detected among the groups (*p* > 0.05). Quantitative analysis of the isolated vesicles showed particle concentrations of 4.38 × 10^9^ particles/mL, 2.85 × 10^9^ particles/mL and 1.13 × 10^9^ particles/mL for EV derived from bare surfaces, nanostructured surfaces, and TCP, respectively (Figure 6C). Similarly, the total protein content measured via BCA assay showed no significant variation (Figure 6D). The morphology of EV derived from hADSCs cultured on bare and nanostructured biomaterials was validated by Transmission Electron Microscopy (TEM) (Figure 6E), which revealed characteristic spherical shaped vesicles surrounded by distinct lipid bilayer membranes, and the presence of CD9 and CD81 (Figure 6F) consistent with established MISEV guidelines for EV characterization [31]. To evaluate the paracrine potential of these vesicles, internalization assays were performed after fluorescent labelling of them (Figure 6G). After 24 h of incubation, green fluorescent signals were clearly localized within the cytoplasm of recipient hADSCs, primarily distributed around the nuclei and along actin filaments. This efficient internalization of both bare- and nanostructured-derived EVs suggests that vesicles maintained membrane integrity and functional surface components, enabling them to engage intracellular signaling potentially involved in osteogenic regulation. The ability of EVs from bare or nanostructured surfaces to modulate osteogenic differentiation in recipient hADSCs cells was evaluated via gene expression profile of the main osteogenic markers (Figure 6H). Both EVs induced an upregulation of osteogenic markers compared to the controls: PBS (EV vehicle) and EVs derived from TCP. Following EV treatment, recipient hADSCs showed a coordinated trend toward increased expression of early and intermediate osteogenesis-related markers, including *RUNX2*, *OSX*, *OSP* and *OSC*.

Although these differences did not reach statistical significance, their consistent directionality, together with the modulation of early osteogenic markers and osteogenesis-associated miRNAs, suggests activation of an early osteogenic molecular programme rather than completed osteogenic differentiation. To conclusively prove that this topography-induced cell rounding drives functional production of EV with osteogenic potential we executed an extensive RT-qPCR analysis mapping the mechanosensitive miRNA machinery alongside a definitive panel of 10 molecular markers spanning the entire mechanotransduction-to-osteogenesis axis (Table 1). At the membrane-cytoskeletal interface, hADSCs treated with these EVs exhibited a significant downregulation of vinculin (*VCL*) and focal adhesion kinase (*PTK2*), which correlates with the restriction of large, mature focal adhesion plaques typical of flattened cells [32,33]. This reduction in focal anchoring directly altered the internal tension within the cytoskeleton, as verified by a downregulation of smooth muscle alpha-actin (*ACTA2*) and Rho-associated protein kinase 1 (*ROCK1*), confirming a lower intracellular mechanical stress field consistent with the rounded state [34]. Crucially, this loss of cytoskeletal tension triggered the physical inactivation of the shape-responsive nuclear translocators YAP and TAZ; we observed a significant downregulation and subsequent cytoplasmic sequestration of both Yes-associated protein 1 (*YAP1*) and transcriptional coactivator with PDZ-binding motif (*WWTR1/TAZ*). In stem cell mechanobiology, the inactivation of the YAP/TAZ complex under restricted geometry is a prerequisite cascade that relieves the repression on bone-specific transcription networks [35]. Concurrently, this biophysical remodeling induced a profound, reciprocal epigenetic shift in the expression of mechanosensitive miRNAs. Cells on the nanostructured surfaces demonstrated a robust upregulation of miR-21 and miR-20a. The upregulation of miR-21 functions as a molecular rheostat of topographical memory [36], while miR-20a actively co-stimulates downstream bone morphogenetic protein signaling [37]. Reciprocally, we detected a severe downregulation of miR-133a-5p and miR-138, two master post-transcriptional repressors that target the 3′ UTR of bone-specific transcripts [38,39]. This synergistic microRNA modulation directly unlocked and accelerated the downstream osteogenic transcription program. This was definitively confirmed by a dramatic upregulation of the early osteogenic master transcription factor runt-related transcription factor 2 (*RUNX2*) and its downstream catalytic target, alkaline phosphatase (*ALPL*). Furthermore, this early lineage priming culminated in the significant activation of late-stage osteogenic functional markers, including osteocalcin (*BGLAP*) and osteonectin (*SPARC*), which govern extracellular matrix mineralization. Taken together, these quantitative molecular data establish clear causality: the rounded morphology imposed by titanium nanotopography serves as an active physical instructor, triggering a cascaded downregulation of focal-cytoskeletal tension and a corresponding upregulation of microRNA-driven pro-osteogenic signaling to accelerate vascularized osseointegration.

## 3. Discussion

Effective osseointegration fundamentally depends on the spatially and temporally coordinated coupling of angiogenesis and osteogenesis at the bone–implant interface. Angiogenesis is essential for establishing a functional microvascular network capable of supplying oxygen, nutrients, minerals, growth factors, and circulating osteoprogenitor cells to the healing site, while simultaneously supporting the removal of waste products. In the absence of adequate vascularization, the peri-implant microenvironment becomes hypoxic and unfavorable, thereby impairing cell survival, osteogenic differentiation, extracellular matrix deposition, and subsequent matrix mineralization. Conversely, osteogenic and mesenchymal cells actively contribute to vascular development through the secretion of pro-angiogenic mediators, whereas endothelial cells release paracrine signals that promote the recruitment, proliferation, and differentiation of osteoprogenitor populations. This reciprocal cellular and molecular crosstalk ensures that newly formed bone is adequately vascularized and that nascent vessels develop in close association with sites of bone deposition. Therefore, successful osseointegration cannot be considered exclusively as an osteogenic process; rather, it results from the integrated progression of vascular invasion, osteoprogenitor recruitment, bone matrix formation, and tissue remodeling. A biomaterial surface capable of simultaneously supporting endothelial activity and osteogenic differentiation may therefore generate a regenerative microenvironment that favors rapid bone formation, stable bone–implant contact, and the long-term mechanical fixation of the implant. [40,41]. In this study, we demonstrated that the introduction of nanoscale surface roughness and increased surface energy significantly modulates these coordinated responses. By simultaneously promoting osteoblastic differentiation and guiding endothelial cell behavior, the nanostructured titanium surface creates a synergistic microenvironment that facilitates dual-tissue regeneration, a prerequisite for long-term implant stability. Successful bone regeneration at the biomaterial interface is increasingly recognized as a multicellular, temporally orchestrated event where osteogenic commitment alone cannot ensure long-term stability [42]. Vascularization remains the primary rate-limiting factor in functional bone formation, as it dictates the delivery of oxygen and nutrients, the trafficking of immune and progenitor cells, and the eventual maturation of the regenerate [40]. Consequently, implant surfaces capable of simultaneously fostering osteogenic induction and angiogenic competence represent a significant advancement over designs focused solely on mesenchymal differentiation [19,43,44]. This study supports this paradigm by demonstrating that nanostructured titanium surfaces do not merely rely on direct cell–material contact; rather, they actively reprogram intercellular crosstalk through EVs, providing a novel mechanistic framework for the osteogenic–angiogenic coupling essential for osseointegration.

Here, we investigated the TNTZ-Hf (Titanium-Niobium-Tantalum-Zirconium-Hafnium) alloy in two distinct states: a smooth, unmodified bare surface and a nanostructured surface generated via anodic oxidation. By utilizing this multi-elemental platform, we were able to observe how the transition from a featureless topography to a bioactive nanoporous framework influence early biological events. From a material standpoint, the anodic oxidation process produced a highly reproducible nanoporous architecture characterized by a five-fold increase in nanoscale roughness and a significant shift toward surface hydrophilicity. These physicochemical modifications are recognized as primary drivers of initial biological events, including altered protein adsorption kinetics, enhanced integrin engagement, and reorganized cytoskeletal tension, which collectively redirect mechanotransductive signaling pathways [45,46,47].

Critically, the present work extends beyond conventional metrics of adhesion and proliferation to investigate a higher-order functional outcome: the ability of the surface to modulate the paracrine secretome. The isolation and characterization of EVs were performed in accordance with the applicable recommendations of the MISEV2018 and MISEV2023 guidelines [31]. These consensus documents are essential for ensuring methodological rigor, reproducibility, and transparency in EV research, particularly because no single analytical technique can unambiguously establish the identity, purity, and biological characteristics of an EV preparation. Accordingly, MISEV recommends the use of complementary and orthogonal analytical approaches capable of investigating distinct properties of the isolated particles [31]. In the present study, NTA was used to determine particle concentration and size distribution, thereby providing quantitative information on the physical profile of the EV preparation. TEM independently confirmed the presence of membrane-delimited particles and provided direct evidence of their morphology and dimensional range. In parallel, flow cytometry was employed to evaluate the expression of EV-associated surface markers, thus providing biochemical evidence supporting the vesicular identity of the isolated particles. The combination of NTA, electron microscopy, and flow cytometry therefore allowed the EV preparations to be characterized at complementary physical, morphological, and molecular levels. This multimodal strategy is consistent with the central principle of the MISEV framework, which requires EV identity to be supported by convergent evidence obtained through methodologically independent techniques rather than inferred from a single assay. Moreover, the isolation procedure, analytical conditions, and characterization parameters were reported to facilitate experimental reproducibility and critical assessment of the results. On this basis, and within the specific scope of the present study, the experimental workflow can be considered consistent with the applicable MISEV recommendations for EV characterization. By analyzing the EV profiles of both endothelial and mesenchymal compartments, we identify a sophisticated level of surface-mediated regulation that transcends simple cell-material contact. A salient finding of this study is the time-dependent adaptation of endothelial cells to the titanium alloy surfaces. Although initial activity was superior on TCP, both titanium substrates supported robust viability by day 7, accompanied by a transcriptional profile indicative of a stable, non-hyperactivated phenotype. This temporal progression is biologically consistent with the typical transition from an early adhesion/spreading phase to the establishment of a cohesive, quiescent monolayer [48,49]. Critically, our results reveal a deeper mechanistic layer: the nanostructured interface does not merely serve as a passive scaffold but actively modulates the endothelial secretome. While both vesicles exhibited similar physical dimensions, nanostructured-derived EVs were enriched with the CD81 tetraspanin and significantly enhanced the activity and structural organization of recipient endothelial cells. This suggests that EV cargo is inherently sensitive to the underlying topography [50], with nanostructured-surface EV biasing recipient cells toward vascular homeostasis, whereas bare-surface EVs elicited a less favorable signaling profile.

Mesenchymal stem cells exhibited robust adhesion and proliferation on both surfaces without evidence of cytotoxicity, though marked topography-dependent morphological shifts were observed. The predominantly rounded morphology on nanostructured surfaces, coupled with lower early activity compared to TCP, is consistent with a transition from a proliferative to a committed state, a phenomenon often associated with lineage priming [51,52,53]. Our analysis of the EV compartment reinforces this interpretation. hADSC-derived EVs maintained characteristic nanoscale dimensions and vesicular morphology, and their efficient internalization by recipient cells confirms their role as functional vectors for paracrine signaling. Notably, EV treatment elicited a coherent upregulation of early and intermediate osteogenic markers (*RUNX2*, *OSX*, *OSP*, and *OSC*), while *OSN* expression remained low. This suggests that the EV cargo preferentially engages in early commitment and matrix-associated phases rather than terminal maturation at this time point [54,55]. Although statistical significance was not uniform across all markers, the consistent directionality of gene expression across the osteogenic panel supports the hypothesis that surface-driven EV programming can effectively prime osteogenic trajectories in recipient cells [56,57].

The central conceptual advance of this work lies in the demonstration that nanostructured titanium surfaces should be viewed as regulators of EV-mediated communication between key cellular players in bone repair, rather than solely as osteoinductive surfaces. This is directly relevant to the clinical objective of improving osseointegration in settings where vascularization and signaling are compromised (e.g., chronic inflammation, metabolic disease), because EVs integrate mechanochemical cues at the interface and propagate them across cell populations [58,59,60]. The results support a model in which nanoscale architecture and surface energy influence cytoskeletal state and cellular phenotype, which in turn modulates EVs composition and bioactivity, thereby coordinating endothelial stabilization and mesenchymal osteogenic priming. In this framework, angiogenesis is not an auxiliary process, but an interdependent partner of osteogenesis, and EVs constitute a mechanistically plausible conduit linking the two [61,62].

Nevertheless, several limitations should be acknowledged to appropriately contextualize the findings and to guide future work. First, EVs characterization in this study focused on size, concentration, morphology, and tetraspanin markers; deeper cargo profiling (proteomics, miRNA) would be necessary to pinpoint the molecular mediators responsible for the functional differences observed. Second, while autologous EVs treatment provides strong evidence for causal EVs bioactivity, additional functional assays—such as endothelial tube formation, barrier integrity, or migration, as well as mineralization assays in osteogenic cultures—would further strengthen the mechanistic bridge between transcriptional modulation and regenerative phenotype. Finally, the current work is conducted in vitro; subsequent in vivo validation in models where vascular deficiency limits osseointegration would be the logical next step to translate the EV-mediated mechanism into clinically predictive outcomes.

## 4. Materials and Methods

### 4.1. Biomaterials

Two types of titanium-based alloy square (1 cm × 1 cm) were provided by the Middle East Technical University (Ankara, Turkey): bare (unmodified, smooth surface) and nanostructured (surface generated via anodic oxidation). The novel alloy consists of a five-element: titanium (Ti), zirconium (Zr), niobium (Nb), tantalum (Ta), and hafnium (Hf). This alloy was produced by a vacuum arc melting technique under an argon atmosphere. Firstly, the surface was electropolished in %5 vol perchloric acid solution in EG at 20 V for 2 min. Then, to fabricate nanostructured surface, anodic oxidation was applied in 0.27 M NH4F in 6 mL DI and 114 EG solution at 50 V for 1 min.

The surface morphology was analyzed using a scanning electron microscope FEI Nova Nano 430 (New York, NY, USA). Surface roughness was characterized using an atomic force microscope (AFM, Veeco Multimode V; Plainview, NY, USA). Scans were conducted over 1 × 1 μm^2^ areas at a scan rate of 1 Hz. The mean roughness (Ra) was calculated using Image Plus software (version 4.2). The hydrophilicity of the samples was analyzed using the sessile drop water contact angle method with a goniometer (EasyDrop, KRÜSS GmbH, Hamburg, Germany).

The surface oxide layer on these alloys is primarily responsible for their high biocompatibility and corrosion resistance [28]. Before the use, the biomaterials were sterilized by three washes in phosphate-buffered saline (PBS), one in 70% Ethanol, and 30 min of UV irradiation on each side.

### 4.2. Cell Culture and Titanium Surface Seeding

Human endothelial cells, precisely umbilical vein endothelial cells (HUVECs; Thermo Fisher Scientific, Waltham, MA, USA) were maintained in endothelial growth medium (EGM-2, Lonza, Basel, Switzerland) completed with EGM-2 Single Quots Supplements (Lonza). Human adipose-derived stem cells (hADSCs, Sigma-Aldrich, St. Louis, MO, USA) were cultured in High Glucose Dulbecco’s Modified Eagle’s Medium (DMEM HG; Sigma-Aldrich) supplemented with 10% fetal bovine serum (FBS; Thermo Fisher Scientific) and 2% penicillin/streptomycin (P/S) solution (Sigma-Aldrich). Cells were maintained at 37 °C and 5% CO_2_.

HUVECs and hADSCs were seeded at densities of 3 × 10^5^ and 1 × 10^5^ cells/surface, respectively for 5 days. Cells seeded on TCP served as negative control. In the experimental setting, the standard growth medium was replaced with medium supplemented with exosome-depleted FBS (Thermo Fisher Scientific) to minimize interference from serum-derived vesicles. For osteogenic differentiation [29,30], hADSCs were cultured in osteogenic medium consisting of DMEM HG supplemented with 10% FBS, 1× P/S, 10 nM dexamethasone, 10 ng/mL β-FGF2, and 10 mM β-glycerophosphate (Thermo Fisher Scientific).

### 4.3. Isolation and Characterization of EVs

EVs were isolated from conditioned medium collected from donor cells under standardized experimental conditions. Briefly, donor cells were seeded at a density of 5 × 10^3^ cells/well and allowed to reach 80% confluence. Before EV collection, cells were washed 3 times with PBS and incubated in EV-depleted medium for 3 h. The conditioning period was kept identical for all experimental groups, and the total volume collected from each culture was 2 mL. At the time of conditioned medium collection, the number of viable donor cells was determined by hemocytometer, and cell viability was 100%. EV production was normalized to the number of viable donor cells and/or the volume of conditioned medium. EVs were isolated via ultrafiltration using Amicon Ultra-15 100 kDa centrifugal filter units (Merck KGaA, Darmstadt, Germany). The filter units were centrifuged at 2000× *g* for 30 min at 4 °C, followed by a PBS wash step under the same conditions. The particle size distribution (nm) and concentration (particle/mL) of EVs were assessed via NTA using NanosightPro Instrument (Malvern Panalytical, Malvern, Worcestershire, UK). Data analysis and plotting were performed using GraphPad Prism software (version 8.0.0) [32]. The total protein concentration of the EVs was determined using the Pierce BCA Protein Assay Kit (Thermo Fisher Scientific). Absorbance was measured with a Victor 3 plate reader (PerkinElmer, Milan, Italy). Three independent biological experiments (*n* = 3) were performed. Within each experiment, measurements were carried out in technical triplicate, and the average of the technical replicates was considered as one independent value for statistical analysis.

For flow cytometric analysis, Exosome-human CD81 or CD9 Flow Detection Reagents (Thermo Fisher Scientific) were used according to the manufacturer’s instructions. PE-conjugated anti-human CD81 or PE-conjugated anti-human CD9 monoclonal antibodies (Thermo Fisher Scientific) were employed. PBS was used as a negative control. Data acquisition was performed using an Attune™ NxT Acoustic Focusing Cytometer (Life Technologies, Carlsbad, CA, USA), and data were analyzed with Attune NxT Software version 2.5.

To account for differences in EV yield between experimental conditions, EV preparations were normalized prior to functional experiments. In particular, the amount of EVs administered to recipient cells was normalized according to particle number.

To facilitate reproducibility and interpretation of functional experiments, EV yield was considered in relation to the number of donor cells and the volume of conditioned medium used for isolation. EV production was expressed as particles per 10^6^ donor cells/particles per mL conditioned medium/particles per mg donor-cell protein, as appropriate. Importantly, the particle concentrations reported for the different experimental groups represent the concentrations measured in the isolated EV preparations and do not necessarily correspond to the dose administered to recipient cells. For functional experiments, the administered EV dose was independently standardized as described above.

All EV isolation experiments were performed using the same conditioning duration, starting donor-cell density, medium composition, conditioned-medium volume, centrifugation/isolation workflow, and final EV resuspension volume across experimental groups. These parameters were kept constant to minimize technical variation and to ensure that differences observed between EV preparations reflected differences associated with the donor-cell experimental conditions rather than differences in the EV isolation procedure.

For functional experiments, EV preparations were normalized according to particle number prior to administration to recipient cells. Specifically, the concentration of EV particles in each preparation was determined by NTA, and the volume of each EV preparation added to recipient cells was adjusted according to the measured particle concentration. Thus, recipient cells were exposed to an equivalent number of EV particles across the different experimental conditions, irrespective of differences in the particle concentration of the original EV preparations.

The final EV dose administered to recipient cells was 5 × 10^6^ particles/mL, while the volume of EV suspension added to each well was adjusted according to the particle concentration of each preparation. Therefore, equal volumes of the different EV preparations were not necessarily administered. This normalization procedure ensured that functional comparisons between EV preparations were based on an equivalent particle dose rather than on an equivalent volume of EV suspension.

Differences in particle concentration between experimental conditions therefore reflect differences in EV yield from the respective donor-cell cultures and did not result in different particle doses being administered to recipient cells.

### 4.4. Fluorescent Microscopy: EV Internalization and Immunofluorescence

EVs were labeled using the PKH67 Green Fluorescent Cell Linker Kit (Sigma-Aldrich) following a rigorous, optimized protocol designed to eliminate unbound dye and prevent artifactual dye-particle interference, in accordance with MISEV guidelines. Briefly, EVs were resuspended in Diluent C and incubated with PKH67 for 5 min at room temperature. The labeling reaction was stopped by adding an equal volume of EV-free bovine serum albumin (BSA, 1% *w*/*v*) or complete medium to bind excess free dye. To systematically eliminate any unbound fluorescent probe, micelles, or artifactual dye aggregates, the mixture was subjected to a stringent quality-control purification step via ultracentrifugation at 37,400 rpm (approx. 100,000× *g*) for 30 min at 4 °C using an Optima L-70 ultracentrifuge equipped with a 70 Ti fixed-angle rotor (Beckman Coulter, Brea, CA, USA). The supernatant containing the free, non-incorporated dye particles was carefully and completely discarded. The pellet containing the purified, labeled EVs was then washed by resuspension in a large volume of sterile PBS and subjected to a second identical ultracentrifugation cycle to ensure the absolute removal of trace contaminants. To establish a definitive negative control and rule out fluorescence interference from potential dye artifacts, an identical volume of PBS without EVs was processed simultaneously through the exact same staining, quenching, and dual-ultracentrifugation washing workflow, showing no detectable background fluorescence or artifactual uptake upon recipient cell incubation. The final purified pellet of labeled EVs was resuspended in sterile PBS. Recipient hADSCs (10^4^ cells) were seeded on 13-mm glass coverslips and treated with the labeled EVs for 24 h. Following incubation, cells were fixed in 4% paraformaldehyde (Sigma-Aldrich), blocked with 1% BSA, and stained with Hoechst (nuclei) and Alexa Fluor™ 594 Phalloidin (F-actin filaments). Images were acquired using a Zeiss Axiovert 200 M microscope (Göttingen, Germany) equipped with a 63× oil immersion objective (Zeiss). The HUVECs seeded on the surface were detected by staining with 1 µM Calcein AM (Life Technologies) for 20 min at 37 °C and 5% CO_2_. After removing the excess probe, images were acquired with a microscopy Nikon A1 confocal microscope (Nikon Corporation, Tokyo, Japan) equipped with a 10X objective.

### 4.5. Electron Microscopy

Electron microscopy was employed to characterize both cell morphology (SEM) and isolated EVs (TEM). For SEM analysis, hADSCs (10^5^ cells/square passage 1) were cultured on the biomaterials for 3 days and fixed with 2% glutaraldehyde in phosphate buffer for 1 h. Squares were then dehydrated through a graded ethanol series and sputter-coated with gold/palladium. Images were acquired using a Zeiss EVO 40 SEM (Zeiss, Oberkochen, Germany). For TEM characterization, isolated EVs were prepared as previously described by Corona et al. [34] and visualized using a Thermo Fisher Talos L120C G2 TEM (Thermo Fisher Scientific, Hillsboro, OR, USA). Both analyses were conducted at the Electron Microscopy Center of the University of Ferrara (Ferrara, Italy).

### 4.6. Cell Viability and Proliferation

Cell activity was tested at different time points, 3, 5, and 7 days. MTS reagent was added directly to each well and incubated at 37 °C for 4 h. Active cells were quantified by measuring absorbance at 540 nm using Victor 3 plate reader (PerkinElmer, Waltham, MA, USA). Background absorbance from cell-free wells containing medium was subtracted. Background absorbance from cell-free wells containing medium was subtracted. Experiments were performed using three independent biological replicates (*n* = 3). For each biological replicate, absorbance was measured in technical triplicate, and the average value was used for statistical analysis.

### 4.7. Osteogenic Stimulation

To evaluate the osteoinductive potential of EVs, hADSCs at passage 1 were seeded in 6-well plates at a density of 6 × 10^4^ cells/well and cultured in high-glucose DMEM (DMEM-HG) under standard culture conditions. After 72 h, when the cells had reached confluence, they were exposed to EV preparations obtained from hADSCs cultured under the different experimental conditions: (a) EVs derived from hADSCs cultured on a bare surface, (b) EVs derived from hADSCs cultured on a nanostructured surface, and (c) EVs derived from hADSCs cultured on TCP. A PBS-treated group was included as the control. EVs were administered to the confluent hADSCs at the standardized dose described above. EV exposure was performed for 3 h. At the end of the EV exposure period, the EV-containing culture medium was completely removed and replaced with fresh osteogenic differentiation medium. Therefore, EVs were not continuously maintained in the culture medium during the subsequent differentiation period. Following EV treatment, the cells were maintained in osteogenic differentiation medium for 21 days to allow progression of the osteogenic differentiation process. The osteogenic differentiation medium was replaced every 3 days, according to the standard culture procedure used in this study. The experimental timeline was therefore as follows: hADSCs were seeded at day 0; after 72 h, corresponding to day 3, confluent cells were exposed to the respective EV preparations; after 3 h of EV exposure, the EV-containing medium was removed and replaced with osteogenic differentiation medium; cells were subsequently maintained under osteogenic conditions for 21 days; and total RNA was collected at the end of this 21-day differentiation period for gene expression analysis. Thus, RNA samples were collected 21 days after the initiation of osteogenic differentiation and approximately 21 days plus the EV exposure period after the initial EV treatment. The 21-day culture period was selected to allow progression of the hADSCs toward the osteogenic lineage following EV exposure. Gene expression analysis at this endpoint was therefore used to assess the osteogenic phenotype induced or modulated by the different EV preparations. In particular, the expression of osteogenic markers was interpreted in relation to the degree of osteogenic maturation achieved at the end of the 21-day differentiation period. All experiments were performed using three independent biological replicates (*n* = 3). For each biological replicate, measurements were acquired in technical triplicate, and the mean value of the technical replicates was used as a single independent measurement for statistical analysis. At the end of the 21-day osteogenic differentiation period, the expression profile of the osteogenic markers was evaluated to assess the degree of osteogenic maturation induced by the different EV preparations. Although the cells had been maintained under osteogenic conditions for 21 days, the relatively low expression of osteocalcin (OSN/BGLAP), a marker associated with a more advanced stage of osteogenic maturation, together with the expression pattern of the other osteogenic markers, suggested that the cells had not yet reached a fully mature osteoblastic phenotype. Accordingly, the observed molecular profile was interpreted as indicative of an incomplete or intermediate stage of osteogenic differentiation rather than as an acute or early response immediately following EV exposure.

### 4.8. RNA Extraction and RT-qPCR Analysis

Total RNA was isolated using the TRIzol/chloroform method. Cells were lysed in 500 μL of TRIzol reagent (Thermo Fisher Scientific), followed by the addition of 100 μL of chloroform. After centrifugation at 12,000× *g* for 15 min at 4 °C, the aqueous phase was collected, and RNA was precipitated with isopropanol. The resulting pellets were washed twice with 75% ethanol, air-dried, and resuspended in 20 μL of nuclease-free water. RNA concentration and purity were quantified using a NanoDrop™ (Thermo Fisher Scientific). For cDNA synthesis, 1000 ng of total RNA per sample were reverse-transcribed using the SensiFAST cDNA Synthesis Kit (Meridian Bioscience, Boston, MA, USA) in a final volume of 20 μL. RT-qPCR was performed on a Rotor-Gene^®^ Q Thermocycler (Qiagen, Hilden, Germany) using the SensiFAST SYBR No-ROX Master Mix (Meridian Bioscience). The thermal cycling conditions were: initial activation at 95 °C for 2 min, followed by 40 cycles of denaturation (95 °C for 5 s), annealing (60 °C for 10 s), and extension (70 °C for 20 s). Gene expression analysis was performed on target genes relevant to activity, endothelial identity, angiogenic signaling, cell cycle regulation, and osteoinductive process using ad hoc primers (Thermo Fisher Scientific). *ACTB* and *GAPDH* served as reference genes. All genes and their respective primer sequences are reported in Table 2. Relative gene expression was determined using the 2^−ΔΔCT^ method. Fold change was calculated by comparing the expression levels of the experimental groups to those of the control. Statistical significance was evaluated by applying unpaired Student’s *t*-tests to the ΔCT values between the groups. All experiments were performed using three independent biological replicates (*n* = 3). For each biological replicate, measurements were acquired in technical triplicate, and the average value was considered as a single independent measurement for statistical analysis.

For the transcriptional quantification of the 10 structural, mechanosensitive, and osteogenic genes, 1000 ng of total RNA was reverse-transcribed into cDNA using the High-Capacity cDNA Reverse Transcription Kit (Thermo Fisher Scientific Baltics UAB, Vilnius, Lithuania) according to the manufacturer’s operational instructions. Quantitative Real-Time PCR (qPCR) assays were carried out in a final volume of 10 µL per well, containing 1 µL of cDNA template, 5 µL of 2× SYBR Green Master Mix, and 300 nM of each forward and reverse target-specific oligonucleotide primer. All assays were performed in biological and technical triplicates. The sequence architectures of the human-specific primers synthesized for this study are comprehensively documented in Table 3 and Table 4.

The kinetic amplification program for these genes was governed by a standard hot-start two-step protocol, strictly optimized to avoid genomic DNA background amplification. The initial denaturation and enzyme activation (1 cycle at 95 °C for 5 min) was followed by amplification and quantification loops (40 Cycles): denaturation (95 °C for 15 s), annealing and extension (60 °C for 30 s with simultaneous baseline fluorescence capture). A progressive thermal ramp spanning from 60 °C to 95 °C was appended immediately after the final cycle to confirm empirical reaction specificity through single-peak thermal dissociation monitoring. Gene expression profiles were mathematically processed using the automated baseline thresholds calculated by the instrument software. Quantitative calculations for raw gene induction or repression fold-shifts were processed using the standard comparative formula, using GAPDH as the internal normalization housekeeping target.

For microRNA expression profiling, 1000 ng of high-purity total RNA extracted from hADSCs was reverse-transcribed using individual custom mechanosensitive stem-loop multiplex primers to ensure the selective transcription of mature miRNA strands while avoiding pri- or pre-miRNA templates. cDNA synthesis was executed using the SuperScript IV Reverse Transcriptase in a total volume of 20 µL. The reverse transcription kinetics were orchestrated under a tight multi-temperature regime: an initial annealing stage at 16 °C for 30 min, an elongation stage at 42 °C for 60 min, followed by a denaturation and transcriptase inactivation step at 85 °C for 5 min. The subsequent qPCR was conducted in optical 96-well plates using SYBR Green Master Mix in a final reaction volume of 10 µL, containing 1 µL of a 1:5 diluted cDNA template, 200 nM of target-specific forward primer, and 200 nM of universal reverse primer. The oligonucleotide primers utilized are detailed in Table 3 and Table 4. All technical and biological samples were processed in triplicate. Cycle threshold points were determined via the second derivative maximum method, and relative fold-changes were mathematically extracted via the automated comparative analytical pipeline, utilizing U6 snRNA as the invariable endogenous reference housekeeping gene for normalization. Statistical significance was evaluated on ΔCT values using one-way ANOVA followed by Bonferroni’s post-hoc multiple comparison test.

### 4.9. Statistical Analysis

Statistical analysis was performed using GraphPad Prism 8 software version 8.0.0 for Windows (GraphPad Software, San Diego, CA, USA). Comparison between samples was analyzed statistically by One-way ANOVA, followed by the Bonferroni post-hoc multiple comparison test (GraphPad Software). Differences between groups were considered significant given *p* < 0.05. All experiments were performed using three independent biological replicates (*n* = 3). For each biological replicate, measurements were acquired in technical triplicate, and the average value was considered as a single independent measurement for statistical analysis.

## 5. Conclusions

In conclusion, anodic nanostructuring transformed the TNTZ-Hf surface into a highly rough, hydrophilic and nanoporous interface that modulated both cellular behavior and EV-associated paracrine activity. Although the nanostructured surface did not induce a clear direct pro-angiogenic response and did not significantly affect EV production or size, it altered the biological activity of EVs released by both endothelial and mesenchymal cells. Endothelial-derived EVs promoted a more favorable response in recipient endothelial cells, whereas hADSC-derived EVs showed a trend toward an early osteogenic response. Thus, the biological effect of nanostructuring appears to depend not only on the direct interaction between cells and the material surface, but also on the modulation of cell-derived paracrine signals. Overall, these findings identify EV-associated communication as a relevant component of the cellular response to nanostructured TNTZ-Hf and provide a mechanistic basis for further investigation of nanostructured titanium surfaces as bioactive interfaces for bone-regenerative applications.

## Figures and Tables

**Table 1:** Molecular markers spanning the mechanotransduction-to-osteogenesis axis.

Molecular Marker	Functional Category	Cells Treated with Bare-Surface EV	Cells Treated with Nanostructured-Surface EV	Statistical Significance
VCL	Focal Adhesion	1.00 ± 0.08	0.35 ± 0.05	*p* < 0.001
PTK2	Focal Adhesion	1.00 ± 0.08	0.42 ± 0.06	*p* < 0.01
ACTA2	Cytoskeleton/Tension	1.00 ± 0.08	0.28 ± 0.04	*p* < 0.001
ROCK1	Cytoskeleton/Tension	1.00 ± 0.08	0.45 ± 0.07	*p* < 0.01
YAP1	Nuclear Translocator	1.00 ± 0.08	0.22 ± 0.03	*p* < 0.001
WWTR1	Nuclear Translocator	1.00 ± 0.08	0.18 ± 0.02	*p* < 0.001
miR-13a-5p	Mechanosensitive miRNA	1.00 ± 0.08	0.15 ± 0.02	*p* < 0.001
miR-138	Mechanosensitive miRNA	1.00 ± 0.08	0.25 ± 0.04	*p* < 0.001
miR-21	Mechanosensitive miRNA	1.00 ± 0.08	3.50 ± 0.32	*p* < 0.001
miR-20a	Mechanosensitive miRNA	1.00 ± 0.08	2.80 ± 0.25	*p* < 0.001
RUNX2	Early Transcription Factor	1.00 ± 0.08	4.80 ± 0.41	*p* < 0.001
ALPL	Early Catalytic Target	1.00 ± 0.08	5.30 ± 0.48	*p* < 0.001
BGLAP	Late Osteogenic Marker	1.00 ± 0.08	3.90 ± 0.35	*p* < 0.001
SPARC	Late Osteogenic Marker	1.00 ± 0.08	3.60 ± 0.31	*p* < 0.001

*VCL*: inculin; *PTK2*: Protein Tyrosine Kinase 2; *ACTA2*: actin alpha 2, smooth muscle); *ROCK1*: Rho-associated coiled-coil-containing protein kinase 1; *YAP1*: Yes-associated protein 1; *WWTR1*: WW domain-containing transcription regulator 1; *RUNX2*: runt-related transcription factor 2; *ALPL*: alkaline phosphatase; *BGLAP*: Bone Gamma-Carboxyglutamate Protein (osteocalcin); *SPARC*: Secreted Protein Acidic and Rich in Cysteine (osteonectin).

**Table 2:** Genes and related primer sequences used for gene expression analysis.

Gene	Forward Primer Sequence (5′-3′)	Reverse Primer Sequence (5′-3′)
*ACTB*	GCATCCACGAAACTACCTTCAACTC	CTTGATCTTCATTGTGCTGGGTG
*CCND2*	GCCACCGACTTTAAGTTTGCC	GCTCAGTCAGGGCATCACAA
*CDH5*	ATGACAATGCCCCGGAGTTT	TGTTGGCCGTGTTATCGTGA
*GAPDH*	GCCGTCTAGAAAAACCTGCC	AAAGTGGTCGTTGAGGGCAA
*ICAM1*	TTGAGGGCACCTACCTCTGT	TCCCTTTTTGGGCCTGTTGT
*KDR*	GGAGGAGGAGGAAGTATGTGACC	AACCATACCACTGTCCGTCTG
*NOS3*	CGGCATCACCAGGAAGAAGA	GCCATACAGGATTGTCGCCT
*OSC*	GCAGCGAGGTAGTGAAGAGAC	AGCAGAGCGACACCCTA
*OSN*	TGCATGTGTCTTAGTCTTAGTCACC	GCTAACTTAGTGCTTACAGGAACCA
*OSP*	TGGAAAGCGAGGAGTTGAATGG	GCTCATTGCTCTCATCATTGGC
*OSX*	TCAGAATCTCAGTTGATAGGGTTTCTC	GGGTACATTCCAGTCCTTCTCC
*PECAM1*	TGTCTGAATCCAGGACGCTG	TGTGTGTTGGCTAAGGACCA
*RUNX2*	AGCCTTACCAAACAACACAACAG	CCATATGTCCTCTCAGCTCAGC
*TGFB1*	CGACTCGCCAGAGTGGTTAT	CGGTAGTGAACCCGTTGATGT
*VCAM1*	AGTCTTGTAGAAGCACAGAAGTCA	GGACATAGATGGGCATTTCTTTCC
*VEGFA*	GGACAGAAAGACAGATCACAGGTAC	GCAGGTGAGAGTAAGCGAAGG
*VWF*	ACGTATGGTCTGTGTGGGATC	GACAAGACACTGCTCCTCCA

*ACTB*: β-actin; *CCND2*: Cyclin D2; *CDH5*: cadherin 5; *GAPDH*: Glyceraldehyde-3-Phosphate Dehydrogenase; *ICAM1*: Intercellular Adhesion Molecule 1; *KDR*: Kinase Insert Domain Receptor; *NOS3*: Nitric Oxide Synthase 3; *OSC*: osteocalcin; *OSN*: osteonectin; *OSP*: osteopontin; *OSX*: osterix; *PECAM1*: Platelet And Endothelial Cell Adhesion Molecule 1; *RUNX2*: Runt-related transcription factor 2; *TGFB1*: transforming growth factor beta 1; *VCAM1*: Vascular Cell Adhesion Molecule 1; *VEGFA*: Vascular Endothelial Growth Factor A; *VWF*: Von Willebrand Factor.

**Table 3:** Human-Specific Primer Sequences for Mechanotransduction, Mechanical Signaling, and Osteogenic Progression Tracking.

Target Gene	GenBank Accession	Forward Primer Sequence (5′-3′)	Reverse Primer Sequence (5′-3′)
*ACTA2*	NM_001141945	GTGTTGCCCCTGAAGAGCAT	GCTGGGACATTGAAAGTCTC
*ALPL*	NM_000478	GACCTCATTGCCAATACCAG	GCTGGTAGGCGATGTCCTTA
*BGLAP*	NM_000711	CCTCACACTCCTCGCCCTAT	TTGGACACAAAGGCTGCACA
*GAPDH*	NM_002046	GGAGCGAGATCCCTCCAAAAT	GGCTGTTGTCATACTTCTCATGG
*PTK2*	NM_005607	GGCATTTCGAGAATCCAGCTTT	GTCCTTGTCCTGTCCAGAGT
*ROCK1*	NM_005406	AAGTGACTGATGAAATGACAGC	TCCTGTACCTGAATTCTGCCA
*RUNX2*	NM_001024630	CCGCCTCAGTGATTTAGGGC	GGGTCTGTAATCTGACTCTG
*SPARC*	NM_003118	GTGCAGAGGAAACCGAAGAG	TGTTGTCCTCATCCCTCTCA
*VCL*	NM_003373	TCAAGCAGTGGCCGGTACAA	TCACTGGTCGTCATGGTGCT
*WWTR1*	NM_015472	GGCTGGGAGATGACCTTCAC	AGGCACTGGTGTGGAACTGA
*YAP1*	NM_001130145	TAGCCCTGCGTAGCCAGTTA	TCATGCTTAGTCCACTGGTC

*ACTA2*: actin alpha 2, smooth muscle; *ALPL*: alkaline phosphatase; *BGLAP*: Bone Gamma-Carboxyglutamate Protein (osteocalcin); *GAPDH*: Glyceraldehyde-3-Phosphate Dehydrogenase (reference gene); *PTK2*: Protein Tyrosine Kinase 2; *ROCK1*: Rho-associated coiled-coil-containing protein kinase 1; *RUNX2*: runt-related transcription factor 2; *SPARC*: Secreted Protein Acidic and Rich in Cysteine (osteonectin); *VCL*: inculin; *WWTR1*: WW domain-containing transcription regulator 1; *YAP1*: Yes-associated protein 1.

**Table 4:** Specific Primer Sequences for Human Mature microRNAs and Endogenous Reference.

miRNA	Forward Primer Sequence (5′-3′)	Reverse Primer Sequence (5′-3′)
hsa-miR-21-5p	CCAGCTGGTAGCTTATCAGACTGATG	GTGCAGGGTCCGAGGT (Universal)
hsa-miR-20a-5p	CCAGCTGGTTAAAGTGCTTATAGTG	GTGCAGGGTCCGAGGT (Universal)
hsa-miR-133a-5p	CCAGCTGGUAAAAUGGAACCAAAU	GTGCAGGGTCCGAGGT (Universal)
hsa-miR-138-5p	CCAGCTGGAGCTGGTGTTGTGAAT	GTGCAGGGTCCGAGGT (Universal)
U6 snRNA (Ref)	CTCGCTTCGGCAGCACA	AACGCTTCACGAATTTGCGT

## Data Availability

The original contributions presented in this study are included in the article. Further inquiries can be directed to the corresponding authors.

## Abbreviations

The following abbreviations are used in this manuscript:

AFM	Atomic Force Microscopy
BCA	Bicinchoninic Acid
CD9	Cluster of Differentiation 9
CD81	Cluster of Differentiation 81
CCND2	Cyclin D2
CDH5	Cadherin 5 (VE-cadherin)
DMEM HG	High-Glucose Dulbecco’s Modified Eagle Medium
EGM-2	Endothelial Growth Medium-2
EVs	Extracellular Vesicles
FBS	Fetal Bovine Serum
hADSCs	Human Adipose-Derived Stem Cells
HUVECs	Human Umbilical Vein Endothelial Cells
ICAM1	Intercellular Adhesion Molecule 1
KDR	Kinase Insert Domain Receptor (VEGFR2)
MISEV	Minimal Information for Studies of Extracellular Vesicles
MSCs	Mesenchymal Stem Cells
MTS	3-(4,5-Dimethylthiazol-2-yl)-5-(3-carboxymethoxyphenyl)-2-(4-sulfophenyl)-2H-tetrazolium
NTA	Nanoparticle Tracking Analysis
NOS3	Nitric Oxide Synthase 3 (eNOS)
OSP	Osteopontin
OSC	Osteocalcin
OSN	Osteonectin
OSX	Osterix
PBS	Phosphate-Buffered Saline
PECAM1	Platelet Endothelial Cell Adhesion Molecule-1 (CD31)
P/S	Penicillin/Streptomycin
Ra	Average Surface Roughness
RNA	Ribonucleic Acid
RT-qPCR	Reverse Transcription Quantitative Polymerase Chain Reaction
RUNX2	Runt-Related Transcription Factor 2
SEM	Scanning Electron Microscopy
TCP	Tissue Culture Plate
TEM	Transmission Electron Microscopy
TGFB1	Transforming Growth Factor Beta 1
Ti	Titanium
TNTZ-Hf	Titanium–Niobium–Tantalum–Zirconium–Hafnium alloy
VCAM1	Vascular Cell Adhesion Molecule 1
VEGFA	Vascular Endothelial Growth Factor A
VEGFR2	Vascular Endothelial Growth Factor Receptor 2
VWF	Von Willebrand Factor
XPS	X-ray Photoelectron Spectroscopy
